# Suicide prevention for the veterinary profession—a preliminary investigation to explore veterinarians’ perceptions of ASIST training for their profession

**DOI:** 10.3389/fvets.2025.1460577

**Published:** 2025-06-02

**Authors:** Judith Smith, Jacinta Hawgood

**Affiliations:** Australian Institute for Suicide Research and Prevention, World Health Organization Collaborating Centre for Research and Training in Suicide Prevention, School of Applied Psychology, Griffith University, Brisbane, QLD, Australia

**Keywords:** workplace suicide prevention, veterinarian suicide, qualitative evaluation, occupational suicide, gatekeeper training evaluation

## Abstract

**Background and aims:**

Several studies have identified that veterinarians are at higher risk of suicide than the general population. To date there has been scant attention to preventing suicide within this profession. Applied Suicide Intervention Skills Training (ASIST) is an evidence-based suicide prevention training program that aims to enhance trainees’ capabilities to help individuals who may be vulnerable to suicidality. ASIST has been successfully delivered to industries and professions where suicide is known to be a high risk and was therefore proposed as a potentially suitable suicide prevention initiative for translation to the veterinary profession. The aim of this study was to conduct a preliminary qualitative evaluation of the ASIST workshop delivered to veterinarians in Queensland, Australia. A secondary aim was to explore veterinarians’ perceived needs for suicide prevention in their profession.

**Method:**

An Interpretative Phenomenological Analysis (IPA) framework was used to explore the experiences of a sample of Queensland veterinarians, after attendance at an ASIST workshop. Participants were females (87.5%) and males (12.5%) aged 30 to 66 years (M = 45, SD = 12.37) who engaged in individual interviews. Thematic analysis identified common and important patterns of meaning within the data.

**Results:**

Three main themes and associated sub-themes were identified: Impactful workshop delivery and learning environment; Relevance of ASIST training for the veterinary profession; and Unique challenges and needs for suicide prevention in the veterinary profession. Overall, participants reported positive experiences of the workshop and its delivery. However, participants also reported feeling a level of discomfort related to some aspects of their participation and made suggestions for tailoring of the workshop to better fit the needs of their peers in future workshop delivery. Participants also suggested important targets for suicide prevention in the veterinary profession.

**Conclusion:**

This study identified key experiences of veterinarians who participated in the ASIST workshop. Important directions for future delivery of the ASIST workshop to the veterinary profession as well as directions for suicide prevention were suggested including actions for improving mental health and well-being in the workplace.

## 1 Introduction

Over 700,000 people take their life every year globally (1) leaving widespread emotional and psychological impacts on those left behind (2, 3), not to mention widespread economic impacts (4). Some occupations have significantly higher suicide rates compared to the general population including agriculture and farming, construction, emergency services (ambulance personnel and firefighters), nursing, transportation, entertainment, and the veterinary health care sector (5–8). Australian veterinarians face a risk of suicide four times higher than the general population (6, 9, 10). Researchers in Norway (11), USA (12) and Australia (13) have identified factors influencing the high rates that are directly connected to work including poor work-life balance, access to lethal drugs, exposure to (animals’) trauma and suffering, pressure to perform, abuse from clients, and moral distress arising from situations when they are obliged to act according to the animal owners’ will even if this is not in the best interests of the patient. Recommendations to prioritize suicide prevention for veterinarians have therefore been made to address suicide in this profession (7). Despite this, scant attention has been given in the literature to design, implementation, and evaluation of prevention activities for them.

A systematic review of suicide prevention in the workplace (14) identified 13 different workplace suicide prevention initiatives (e.g., targeting or addressing construction, transport, farming) (14) but found a dearth of information about their effectiveness. However, of the small number of evaluations undertaken, two demonstrated reductions in the number of suicides over an 11- and 13-year period, and one found a reduction in suicidal ideation after a two-year period (14).

Importantly, the suicide prevention initiatives associated with this positive impacts/effectiveness included some of the key recommendations of the World Health Organization (WHO) (14, 15). WHO have recommended suicide prevention approaches that include a wholistic approach where multiple initiatives target multiple levels within the workplace to boost protective factors (which buffer against adversity or risk factors) and mitigate risk factors for suicide (15).

MATES in Construction (MATES) mental health and suicide prevention program (16) has been recommended by the WHO as a successful example of building capacity for workplace suicide prevention (15). The MATES model is a multi-level, industry-based and workplace approach to suicide prevention. It is inclusive of general awareness training (GAT), a 1-h educational session about suicide literacy and aimed at all workers on a given (construction) site; Connector training, 4-h long, and based on the LivingWorks training program SafeTALK, which teaches peers (within the industry) to connect with and respond to signs of distress in their peer workers by providing further support; and, the more intensive 2-day ASIST program, which is more in-depth and focused on intervention—developing a safety plan and supporting people to identify other supports or resources to assist them. Evaluations of MATES as a whole, and for separate training programs, have found evidence for effectiveness in reducing suicide risk, and improving mental health, suicide prevention literacy, peer helping intentions, and reduction of stigma (17).

LivingWorks ASIST is a leading suicide interventions skills training program, globally recognized by the World Health Organization (18). ASIST is a 2-day gatekeeper training (GKT) that aims to build competencies of people to act as helpers (commonly referred to as gatekeepers) to identify signs of suicidal distress in individuals and refer them to help (18). In the general literature on GKT, ‘gatekeeper’ is the term denoted for trainees who complete GKT; however, the preference of LivingWorks is for ASIST trainees to be referred to as helpers (rather than gatekeepers). ASIST has four intermediate outcomes including: (a) identification of risk, (b) connecting, (c) understanding, and (d) assisting (19). Given that the ASIST program has been successfully adapted and applied within the construction industry (16), and in recognition of the high suicide rates in veterinarians, it was proposed by a veterinarian with knowledge of ASIST (who is also an ASIST trainer), that veterinarians may be best placed to identify their peers in distress and provide immediate support and safety by referring them to help. However, to date, there is no evidence for the suitability of ASIST within the veterinary profession; especially as perceived by veterinarians themselves.

This study aims to address this gap and explore the experiences and perceived suitability of the ASIST workshop for veterinarians, as well as their perceptions concerning the need for suicide prevention in their profession.

## 2 Method

A qualitative approach was employed using an IPA framework (20) to gain rich insights into the personal experiences and perspectives of Australian veterinary professionals following participation in the 2-day face to face ASIST workshop in July 2023. IPA is a research design that offers an opportunity for a story to unfold by encouraging participants to express freely their subjective interpretation of their own experiences. The researcher then searches for and identifies themes within the narratives (20). Phenomenology is the study of the nature of people’s experiences when they experience a phenomenon (21). Thus, IPA was considered the most appropriate interpretive approach for this study as it allowed many viewpoints of the phenomenon that each were true from the experience of each participant; rather than seeking an absolute truth about the phenomenon itself (22). Furthermore, given the exploratory nature of the study, we were specifically keen to understand veterinarians’ experiences of the workshop as opposed to quantifying numerically the workshop impacts (as in quantitative approaches to training evaluation).

### 2.1 Participants, procedure and design

The study adhered to Consolidated Criteria for Reporting Qualitative Research (COREQ) (23) which informs both rigor and ability for subsequent replication of the study. Ethical approval was gained from the Griffith University Human Research Ethics Committee (GU Ref No: 2023/393). The researchers were approached to evaluate the ASIST workshop for veterinarians, which was formalized through a connection between the Australian Institute for Suicide Research and Prevention (AISRAP, Griffith University) and LivingWorks. The workshop itself was facilitated by LivingWorks which followed the standardized ASIST format of a two-day in-person delivery mode. The researchers were not present at the workshop. Participants were veterinarians and veterinary nurses (*N* = 8) who had been invited to attend by LivingWorks. Facilitators of the workshop were trained in safeTALK Training for Trainers (24). One trained facilitator was also a veterinarian. All workshop participants voluntarily engaged in individual interviews of approximately 45 min duration, conducted by the lead author (J.S.). Interviews occurred within 2 to 4 weeks after the workshop.

### 2.2 Data collection

The interview schedule included a series of open-ended questions commencing with broad and moving to more probing and guiding questions as the interview progressed, allowing participants to respond at length in their own words reflecting their individual experiences. For example, study aim one was addressed by asking the lead question: “What was your experience of the training?” while the lead question for study aim two was: “What do you see as the biggest areas of need regarding suicide prevention in the veterinary occupation/community/profession?”

Individual interviews were conducted by the lead researcher who also recorded notes immediately after the interviews. Microsoft TEAMS was used for scheduled virtual interviews at a date and time convenient to the participant within the two-to-four-week period after the ASIST workshop. All interviews were recorded with participant permission and the transcription function within the TEAMS recording was used to provide a transcript of each interview. All interviews were checked to ensure accuracy of verbatim in text. Participants were offered the opportunity to choose a pseudonym to be used to provide maximum anonymity for reporting of their narratives in the study outcomes.

Demographic data collected at the time of the interviews included age, gender, length of time employed, qualification, role, type of practice (e.g., small or large animal), and current employment status.

### 2.3 Data analysis

We adopted an inductive approach for understanding the experiences of participants. Following downloading of transcribed interview data, the lead researcher (J.S.) listened to the interview recordings to correct any anomalies in the transcriptions. The transcripts were then read and re-read by both researchers (J.S., J.H.) to ensure familiarity with all interview content, and transcripts were copied and pasted into an excel document ready to be coded, analyzed, interpreted and verified. Thematic analysis was used to analyze the data. Both researchers became familiar with the data by reviewing the excel documents against transcripts, and then proceeded to conduct manual analysis to identify meaningful statements within the data. Salient sections of data were highlighted and meaningful notes taken by both researchers, each in a dedicated column of the excel spreadsheet for later comparison. Both researchers journaled their thoughts, reflections and responses to the data throughout the entire process to ensure integrity of the research. NVivo 12 software (25) was also used to sort and arrange the data into proposed themes and subthemes.

### 2.4 Rigor

Rigor and trustworthiness of data analysis was informed by and in line with the COREQ guidelines for conduct of qualitative research. To enhance reliability of the entire coding and analysis process, the second author (J.H.) was responsible for coding half of the interview data (for example, all of the even-numbered interview transcripts for participants 2, 4, 6 and 8). J.S. coded and analyzed *all* interview data. After undertaking the coding of data independently, J.H. and J.S. attended several meetings to compare notes made against the raw data, and to summarize statements for extracting meaning from the data. Subsequently the researchers applied meanings and grouped the data into thematic clusters to identify common and important concepts. Again, the parties worked independently first and then came together to reassess all themes and interpretations. Any discrepancies were noted and negotiated until consensus was reached. This iterative revision process was used to create the final list of themes. Data saturation was reached after eight interviews. Both researchers identified quotes and extracts from the data that most vividly showcased the outcomes of the research.

The lead researcher (J.S.) is a Master of Suicidology alumni who was undertaking the study as part of her dissertation. She has a background in suicidology and business administration and is passionate about mental health and wellbeing in the workplace. Her interest in veterinary wellbeing began in a conversation with a veterinarian about aspects of their personal experience that led her to hope to support the profession from her standpoint as a suicidologist. The second researcher (J.H.) is an experienced clinical psychologist and researcher of 25 years and has conducted numerous qualitative studies in suicide related domains. J.H. was also the supervisor of the lead author as part of the dissertation component on the Master of Suicidology qualification. The researchers had no prior relationships with participants of the study who were also members of the ASIST workshop.

## 3 Results

### 3.1 Demographics of participants

While 10 participants from the total pool of workshop participants volunteered originally to participate in interviews, only eight people were able to attend on the day of their scheduled interview due to logistical reasons. Participants were aged 30 to 66 years (M = 45, SD = 12.37). Four were qualified veterinarians which, in Australia requires a Bachelor of Veterinary Science degree (BVSc), and four were qualified veterinary nurses (Diploma/Cert IV); two had additional qualifications in counseling, and one was undertaking a degree in psychology as an additional qualification. All were working; four part-time and four full-time (see Table 1 for details).

**Table 1:** Demographics of participants.

Characteristic	*n*	*%*	Range	Mean	Median	*SD*
Relevant qualification						
BVSc (Bachelor of Veterinary Science)	4	50.0	-	-	-	-
Diploma (Veterinary surgical nursing)	1	12.5	-	-	-	-
Cert IV (Veterinary nursing)	3	37.5	-	-	-	-
Additional qualification (counseling)	2	25.0	-	-	-	-
Additional study (psychology)	1	12.5	-	-	-	-
Years working	-		10–45	24	20.5	11.02
Age	-		30–66	45	43	12.37
Type of practice						
Small animal	6	75.0	-	-	-	-
Exotics	1	12.5	-	-	-	-
Mixed / rural	1	12.5	-	-	-	-
Current workload						
Full time	4	50.0	-	-	-	-
Part time	4	50.0	-	-	-	-
Gender						
Female	7	87.5	-	-	-	-
Male	1	12.5	-	-	-	-
Total participants	8					

### 3.2 Themes

Thematic analysis revealed three main themes from the participant interviews: (1) Impactful workshop delivery and learning environment; (2) Relevance of ASIST for the veterinary profession; (3) Unique challenges and needs for suicide prevention in the veterinary profession.

**Figure 1:**
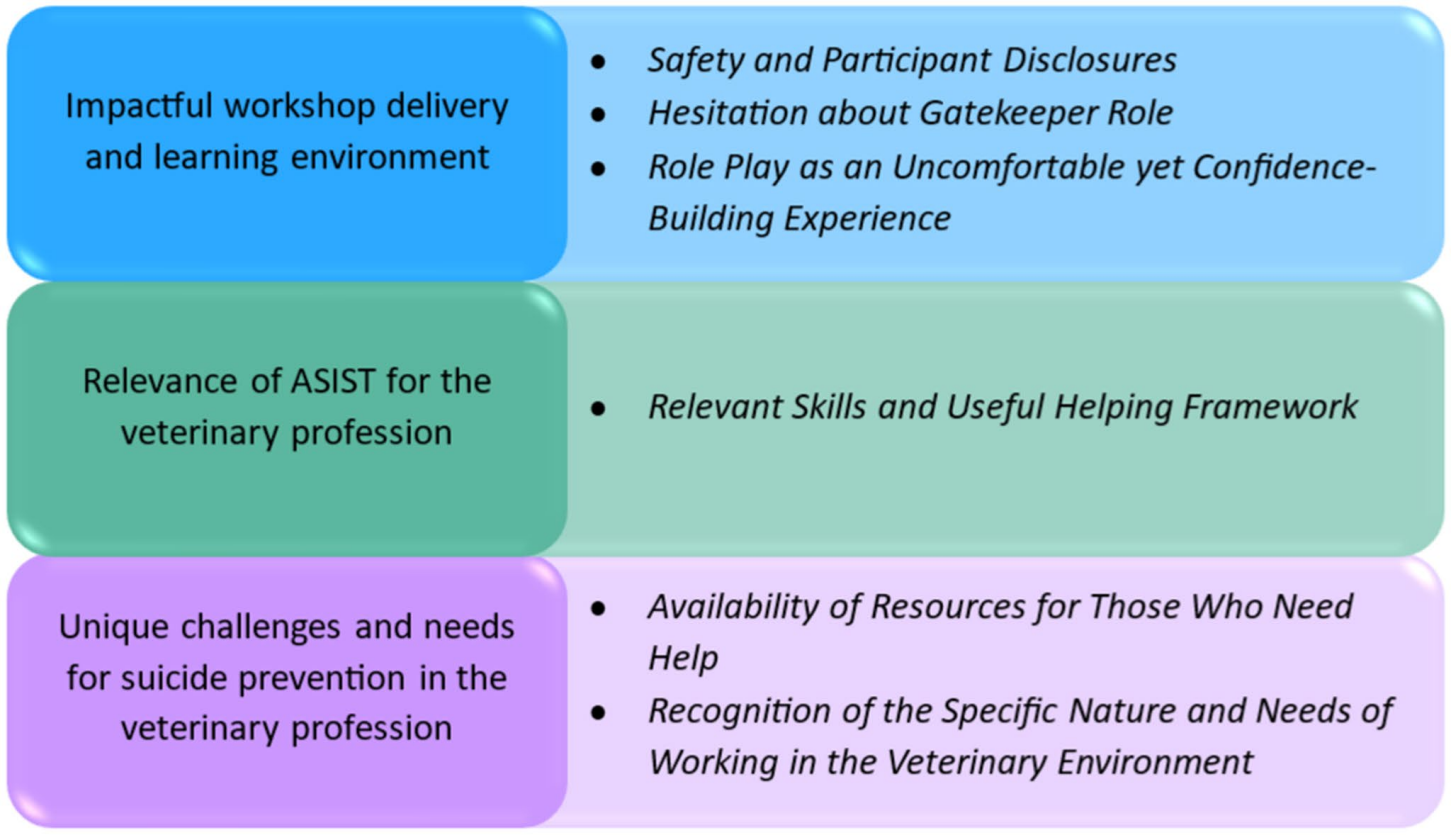
Summary of themes and sub-themes.

As can be seen in Figure 1, several sub-themes were also identified under each key theme. The themes and subthemes are described below in detail, supported by verbatim quotes extracted from the interview transcripts.

#### 3.2.1 Impactful workshop delivery and learning environment

All participants reported that the workshop was delivered well, with positive feedback about the mindful approach by trainers and the safe environment which facilitated openness among participants. Participants reported experiencing a range of emotions during the workshop; both positive and negative. Participants felt enthusiastic about their learning and connected among their peers in the shared prospect of helping to save lives. However, they also felt confronted by the open natured discussion of suicide and discomfort about participating in practice sessions, although they felt safe and supported by the trainers and their peers generally.

##### 3.2.1.1 Safety and participant disclosures

Several participants described the experience of feeling confronted, surprised and sad when experiencing the openness and vulnerability of their fellow veterinary participants sharing personal, ‘suicide related’ experiences in the workshop:

*Stella:* On the first day particularly, I was taken aback at how confronting it was. So, when we separated into our two groups and they asked us how suicide has been [present] in your life, … I kind of thought it was casual conversation… But people went quite deep into it. And I probably wasn’t expecting that. Like some of them [spoke] about themselves, some of them about other vets and family members and stuff [they are] going through now and that was quite emotional.

Many participants shared personal experiences of their own or of their immediate workmates, which they reported was easier to do among peers who understood the profession:

*Mog*: Having it catered to different industries, I think that’s really important.

That way … we all sort of knew where each person was coming from. [A lot of peoples’ personal stories] were related to the stresses that the veterinary industry, you know, puts on us. I think it was just nice to sort of be amongst your own folk talking about it.

Participants felt that sharing their experiences in the workshop among peers validated their own experiences and led to deeper insights. Despite the vulnerability of exposing what are often hidden truths, “it was presented in a really mindful, sensitive way; they [the trainers] were really sort of aware of keeping everyone in the training safe, which I really appreciated,” (Molly). Other participants also commented that they felt emotional yet also scaffolded by the trainers’ experienced approach:

*George*: [It was] emotional because we were reflecting on our experiences with suicide, which is, you know, very charged, [an] emotionally charged event. You know when you have come across those experiences in your life, you know, and I think … we are all feeling that intensity of that… it was quite an emotional roller coaster I would say.

Despite participants’ reflections about feeling safe with disclosures, this feeling appeared to take time to develop. At the start of the workshop, some participants felt confronted by the discussion. Participants came to the workshop with awareness of the rates of suicide among veterinarians: “we all know the stats,” (Stella), but the fact that “basically everyone in the room [knew] someone that they closely work with [who had experience of] suicide” (Stella), and the depth of disclosures, brought to the fore a sense of the closeness and immediacy of the problem:

*Emma:* I felt that it was quite confronting to start with when, not so much the training itself, but at the beginning, everybody sort of sat down and discussed um… their sort of personal experience with suicide, whether that was like colleagues or family members or what not. And I think that was quite confronting … not in that the discussion was about suicide, but more so that it was such a prevalent sort of experience.

##### 3.2.1.2 Hesitation about gatekeeper role

Participants expressed feeling hesitant about taking on the suicide prevention gatekeeper (helper) role despite their expressed support for helping their peer veterinarians in distress. There was also some concern expressed about what the helper role would look like in the context of their work role and what impact it would have on them personally, and their colleagues:

*Emma*: You know, you are trying to help them, but you can only do so much. And obviously you do not wanna take too much on and then negatively affect yourself as well when you are not a professional and you probably cannot help them in all the ways that they need.

##### 3.2.1.3 Role play as an uncomfortable yet confidence-building experience

Participants reflected on how they felt during the practice sessions (or role plays), describing an initial level of discomfort associated with participating in them. Some commented that they had reservations about participating but were not given an option to not participate:

*Emily*: And I was okay with doing role plays, but if people had not been, then I think they would have, you know, struggled and it would have been nice for people to have an option. But they did not say [that] the role play is, you know, optional. You do not have to do it. It was kind of just assumed that you’d do it and you’d be okay with it.

Some participants reported feeling initially uncomfortable about using the word suicide out loud, but all reported that asking the question [are you having thoughts of suicide?] in the practice sessions increased their confidence and reduced their own hesitations about having conversations about suicide. For example: “It still feels like there’s a bit of a taboo around mentioning it, and I feel like the more we talk about it, the easier it becomes to talk about it. And that’s half the problem.” (Molly); “It just left me feeling far more confident to approach a situation. Whereas before I would have been very reserved about it.” (Molly); “[I am] confident that I can have that difficult conversation with someone and know how to walk through it … I’ve come away with the skill set to be able to action that conversation.” (Louise).

#### 3.2.2 Relevance of ASIST training for the veterinary profession

Participants spoke highly of the ASIST training. They felt understood and encouraged by experiencing the training with their peers. They also described both the relevance of the training for them and ways in which the training could be even more impactful if tailored to their unique workplace experiences.

##### 3.2.2.1 Relevant skills and useful helping framework

Participants described their experiences of discovering new insights, gaining “a different perspective on how we talk about suicide” (Mog). A participant conveyed satisfaction with how ASIST helped them to translate newly acquired knowledge into practical skills, referring to “good practical advice about, you know, how to really approach someone that might have been expressing suicidal thoughts” (George). This type of satisfaction was also experienced by those who reported having prior training in mental health:

*Stella*: I’ve done the mental health first aid before, which sort of, I guess more focuses on a general mental health crisis and there [are] parts about suicide, but not in depth, I guess. I think the ASIST program is really good.

Participants reported having used the newly acquired knowledge and skills since the workshop and positively described the ASIST framework:

*Louise*: Pretty much since I’ve done the course, every person that I have had contact with and that’s a lot of people, …I’ve been having conversations about this program and being like, ‘You need to do it’ because I think it’s removing that stigma that the conversation should only be had with a manager or with the HR department when that’s not true.

#### 3.2.3 Unique challenges and needs for suicide prevention in the veterinary profession

Participants’ discussion of the benefits of the ASIST workshop led to identification of several areas of need associated with their future role of being a ‘gatekeeper’ within the veterinary profession. Two main subthemes were identified: availability of resources for those who need help; and recognition of the specific nature and needs of working in the veterinary environment.

##### 3.2.3.1 Availability of resources for those who need help

Most participants expressed concerns about their limited understanding and lack of knowledge about the nature and quality of available resources as the next stage of getting actual help for a person in a crisis. As gatekeepers, they did not feel sufficiently informed as to which gate to recommend, and what would be found on the other side:

*Stella*: I found the keeping them safe situation helpful, but I did wonder, from there, I felt like there’s a little bit of pressure to take that on yourself … I understand that keeping them safe was like, can we get you through 24 h … but from there I felt a little bit lost as to where to go.

*Emma*: I think that ASIST definitely can play a really valuable role in, you know, me buying a little bit of time, so to speak, but I think at the end of the day the professional system needs to be what supports them on a more of an ongoing basis. And I think that accessing that seems to be incredibly difficult. So, I think whether it’s making sure that the ASIST training has a more clear sort of integration with the next step … because I felt that was the least clear part of the whole process.

Participants who did have some knowledge of, or experience with, some of the available support systems in their general community expressed concern, frustration and fear about the quality and availability of this support. For example, one participant referred to comments made by those in the workshop:

*Stella*: Some of them [were] talking about reaching out to some of the resources that are like widely recommended and not getting much from it and one of them being … the EAP [Employee Assistance Program] … one person said that they actually reached out to them in a time of desperation and were told: ‘Yeah, that’s cool. We’ll make you an appointment next week.’ … I did not realise that you did not just call up and talk to someone immediately. … that person has made a big call to … actually dial that phone number expecting, you know [to receive immediate help].

And another participant commented on the limited amount of information provided in the workshop concerning connecting people with help:

*Emma*: The connection to professional support avenues just wasn’t explained in enough detail for me personally. I think that knowing what to do after you have got this person to sort of admit they are suicidal, you have made an immediate plan as to what to do next, but then where to go from there? … They do need, like anyone in this position needs, professional sort of support and trying to find, [to] navigate through the mental health support system is not easy.

##### 3.2.3.2 Recognition of the specific nature and needs of working in the veterinary environment

When asked about their views concerning suicide prevention for the veterinary profession, nearly all participants emphasized the importance of acknowledging the unique profession-related needs of veterinary work and the need for “recognition that it is a high stress industry; there are a lot of challenging interactions and high emotional situations” (Emma). Specifically, they described the complexities, challenges and emotional nature of the work that they do:

*Louise:* The veterinary industry is so intense, and it is really, it’s a very draining industry because it’s so emotive, you know you like, you have got the emotions of the client you have got, your emotions to do with the welfare of that pet, you have got the emotions of your other team members that come into play and you know, especially when you are dealing with traumatic situations.

Some participants highlighted the negative treatment by clients toward veterinary support staff versus veterinarians noting that “the way clients treat nurses is completely different to how clients treat vets. So, clients are more inclined to be really rude to nurses and disregard our recommendations or professional advice, and treat us terribly” (Louise). Molly also stated that “we have had clients threaten suicide” but that following ASIST “my anxiety around that would be far reduced.”

Some participants highlighted the traumatizing nature of experiences unique to veterinarians and their support staff, such as performing euthanasia.

*Louise*: You know the nurse or the receptionist is the one who takes that initial booking [for a pet to be euthanised], it’s the nurse who takes them into the room and gets them settled and you know, gets them to sign the consent paper to put their pet to sleep, which for a lot of people is a huge thing to sign, that piece of paper … and the nurse stays in the room and we will you know, wrap the pet up in its favourite blanket and get them already to be taken home or go through the process [organising] for Fluffy to be picked up by a cremation company and we then support those clients as they leave the clinic and you know it is highly emotional.

Others spoke about the need to develop resilience skills to enable them to deal with the inevitable heart-wrenching experience of an animal patient’s death:

*George*: Some of these students, they are very clever young people and you know, they have been extremely successful in their academic careers and then to suddenly, you know, encounter failure where things die and people cry and it’s just humiliating or humbling … and they need obviously sometimes … support. When you when you fail, you need to know that that’s OK, that’s part of life that you have to get up and forgive yourself and … hopefully learn from whatever happened and get back up on your feet and be a vet for another day.

Several participants referred to negative workplace conditions as being a key target for address in the context of suicide prevention. Specifically, they noted not being able to take breaks and financial conditions as being related to distress that could be prevented: “Most days we do not get a lunch break. It’s rare to get an actual break, that sort of thing. So, I mean, even if they started with a lunch break. And… you know, a reasonable pay” (Emily) and low pay: “So, I mean, you might do a surgery that’s worth $2000 and it will take you an hour and you get $60 of that $2000” (Emily).

*Louise*: You get paid more money to work at ALDI than you would to be a vet nurse and honestly like, when I reflect upon my own skill set, I think it is phenomenal the amount of information and critical knowledge that I have regarding so many different species … and nurses are getting second jobs because our rate of pay is so menial to our, to the skill set that we have.

Participants commented that due to the specific nature of events that are likely to cause distress in their profession, talking to counselors from “outside their world” may only have limited benefit, including an Employee Assistance Program that has been created to support them.

*George*: The AVA [Australian Veterinary Association] has a counselling service. Um, yeah. My criticism of that you are only going to connect to a generalised counsellor and I’ve being personally trying to correlate counsellors that have veterinary specific experience. So, I think to increase the quality of the counselling there will really help.

Participants also reported a desire for increased access to suicide prevention training: “[with training], I feel like every member of staff needs to have the ability to have the conversation and see those invitations,” (Louise), as well as a “continuous focus on mental health” (George), conveyed as a need to be proactive rather than reactive—as endorsed further by Louise: “I suppose having a constant level of focus, you know, not a ‘We’ll look at it now because we think that there’s a crisis with, you know, suicide.’ But then people get distracted by other things and the focus goes to other areas.”

Some participants also spoke about suicide among veterinary nurses, and their perception that the support staff need, but may not receive, as much support as veterinarians:

*Louise*: Yes, there is a huge issue with vets taking their own lives, but the issue goes beyond that. It’s nurses, it’s kennel hands, its receptionists, you know, it’s not just vets. I feel like the focus needs to come off it just being a vet-related issue because … I suppose in the media … they sort of say you know it’s the vet suicide rate but it needs to be the vet industry suicide rate you know because I know just as many, if not more, nurses and support staff who have taken their own life as I have vets.

They also spoke about the positive effects of corporatization on the profession but noted a downside to this was a perception that they were seen as “money earning units, productivity units” (Emily), whereas “[it would be better] having management and company executives look at clinics as a bunch of humans who are trying their absolute hardest, instead of putting a dollar value on everything” (Louise). Mental health initiatives that were provided were perceived by some as more for show than effect: “You know, so they pay lip service to it … they say they support mental health, but when it comes to it, you know, you do not feel supported” (Emily). Others spoke further of this perceived lack of support for individual workers:

*CK*: I think corporatisation has brought some benefits, but … they are not really aware of what goes on in clinic. They’re just kind of looking at these, you know, production statements … It’s not that sort of an industry. You know anybody who’s in it loves animals, loves what they do and they just wanna do the best they can anyway. So, you do not have to push them to do a good job.

## 4 Discussion

This is the first qualitative exploratory evaluation of ASIST for veterinarians in Australia. Two key themes were identified in response to the primary study aim to explore perceptions of the ASIST workshop for the veterinary profession: Impactful workshop delivery and learning environment; and Relevance of ASIST training for the veterinary profession. A third main theme was derived from the secondary study aim to gain insights into veterinarians’ perceptions of what is needed to prevent suicide in their profession: Unique challenges and needs for suicide prevention in the veterinary profession.

### 4.1 Impactful workshop delivery and learning environment

This theme emerged across all participant interviews, as participants described the profound and enduring impact that the ASIST workshop had on them. The sub-theme of Safety and participant disclosures highlights the impact that the delivery elements and training environment had on participants in addition to the content itself. Participants attributed their peers’ willingness to be open and vulnerable to the safety element of the workshop environment, created by the trainers. It is not uncommon for participant disclosures of suicidality to surface during training when the environment is perceived as safe to do so (26). Lived experience is valuable in suicide prevention as it broadens perspectives and provides deeper, more ‘real-world’ expert insights into what is experienced and how best to respond (27). People with lived experience are uniquely positioned to provide understanding without judgment or fear-based responses (28). Previous evaluations of ASIST have identified learning from the experiences of others as a valuable part of the training, especially among participants who were not from a mental health background (29). These findings highlight the importance of considering the delivery elements when developing and delivering training to an audience that is not usually engaged in the suicide prevention sector.

The practice sessions were perceived by some participants as uncomfortable, yet confidence-building. Overall, the practice sessions were also considered pivotal for overcoming discomfort. Participants perceived that rehearsal of direct and sensitive use of this language would assist them in the ‘real world’ conversations. Evidence in support of ASIST training methods has shown that practice sessions and small group work improves group cohesiveness, comfort and enhances learning (30). Given that veterinarians do not engage in suicide-related helping behaviors in their day-to-day professional roles, future ASIST workshops might benefit from preparatory elements to enhance participant readiness for engaging in practice sessions which may initially be experienced as challenging.

Participants were hesitant about taking on the suicide prevention gatekeeper (helping) role, despite their motivation to do the training. This experience appeared to be related to concerns about lack of clarity and knowledge about what the role would look like; specifically, with regards to obtaining help for a person in suicidal distress. The literature does not appear to have explored this issue experienced by gatekeeper trainees in either existing ASIST evaluations nor from any other type of GKT study. This is despite accounts in the literature more generally around the experience of fear and anxiety in workers in suicide prevention; commonly associated with lack of suicide prevention training, reduced knowledge and lower confidence in intervention (31).

### 4.2 Relevance of ASIST training for the veterinary profession

This theme was dominated by participants’ high level of perceived benefit of a tailored profession-specific workshop. Participants felt a sense of safety in their learning enhanced by the knowledge that they were among peers. This overall finding is consistent with other evaluations of ASIST when delivered to industry specific groups, e.g., MATES in Construction (17), where construction workers have reported that they placed the highest trust in their peers for confiding mental health concerns and seeking advice. All participants conveyed the sentiment that others from outside their peer group may not understand the nature of their experiences. The fact that all participants were from the veterinary profession enhanced the intimacy, safety, and level of understanding that was shared by the participants.

The sub-theme, Relevant skills and useful helping framework, best depicts the continual emphasis by participants on how the ASIST workshop enhanced their abilities for picking up or noticing warning signs in others in distress, and how helpful it has been for facilitating open conversations about suicidality and providing support and encouragement for their peers to access/reach out for help. Some participants had previously engaged in suicide prevention related training (e.g., counseling or mental health first aid), yet they perceived that the ASIST workshop provided additional and unique skills, insights and enhanced knowledge. Participants reflected on how the concept of an ‘invitation’ was helpful to identify meanings behind what people say while the ‘turning point’ was useful for identifying when the person had become willing to engage with help; finally, ‘safe for now’ was experienced as a new concept beyond ‘doing’ or ‘not doing’ anything and provided a space in which they could engage and attempt to help them consider other options. This focus in delivery of learning ‘safety’ skills as opposed to risk mitigation or over-cautious reactions is unique to ASIST (32). These findings are important as prior evaluations of ASIST have quantitatively measured the useful elements or outcomes of the training (e.g., confidence and/or knowledge in broad areas, like intervention and referral) (19); however, the current qualitative study provides rich and more specific examples of the skills acquired and applied. Further, these findings provide evidence of positive uptake of this approach, which speaks to a helpful element in this training workshop for veterinarians.

### 4.3 Unique challenges and needs for suicide prevention in the veterinary profession

The final theme of the study was dominated by a sense of uncertainty but also some clarity about the needs of veterinarians with regards to suicide prevention in their profession. With respect to availability of resources for those who need help, some participants shared experiences about their own or their colleagues’ attempts to get help in the past that had been inadequate or unsuccessful. This supports the finding by Connolly et al. (33) that there can be substantial barriers to obtaining help for veterinarians who seek it. Veterinarians who overcome perceived stigma and excessive workloads to find willingness and time to seek help often meet other practical barriers such as insufficient availability of practitioners, especially in rural areas (33). Predominantly, the findings pointed to a fear of being out of their depth in trying to help, which went hand in hand with the heightened awareness that to act confidently in the role of gatekeeper would require more information about referral outcomes.

Recognition of the specific nature and needs of working in the veterinary environment naturally reflected participants’ heightened motivation to want to prevent suicide in their profession. Resilience was raised as an important skill for veterinarians due to the inherent risk of psychosocial stressors in their workplace. Recent studies (34, 35) suggest that resilience and coping skills can be enhanced by building a healthy work environment, personal and social skills training and promoting social support among peers. There was a sense of helplessness to optimally address suicide prevention given the more pressing issues of their working environment. For example, participants spoke of a vet shortage as a contributor to excessive work demands. Workload and long hours of work had already been identified as a stressor in studies prior to 2020 (10, 36–38) and this has intensified with the increase in pet ownership during and since COVID-19 (13, 39, 40). Pet ownership in Australia increased from 61% of households having a pet in 2019 to 69% in 2021 (41). The demand for veterinary services is also expected to increase with future growth of 19.7% projected for the veterinary job market from 2021 to 2026 (42). For most participants the effects of the vet shortage are being felt in terms of increased pressure to work harder, faster and longer because there are not enough people sharing the load.

The discussion of the nature of work also highlighted perceptions of poor workplace conditions and low rates of pay which are in stark contrast to the high cost of veterinary care perceived by pet owners. The relationship between work-related problems and suicidal behavior has been validated by the findings of the NORVET study (11) on veterinarians which reported workplace factors (including emotional demands, work/life balance and fear of complaints/criticism) as the most significant contributor to suicidal thoughts. Specifically, of those participants reporting suicidal thoughts (*n* = 135), nearly 50% (*n* = 65) identified ‘work problems’ as a major contributor to those thoughts. This is also consistent with the finding of Platt et al.’s (38) systematic review which identified 12 occupational difficulties for veterinarians including long working hours, workload, client demands/expectations, work-life balance and euthanasia among the stressors for veterinarians. Of 52 studies reviewed, 36 mentioned at least one of the identified workplace issues (38). Unrealistic work expectations and impersonal management prioritizing financial outcomes of the job are also consistent with the findings in the AVA Wellness report (13). This report outlined that participants experienced poor work-life balance and situations where it was impossible to take breaks or complete necessary tasks in order to leave work on time.

Despite veterinarians in the current study reporting not needing to be motivated by targets, key performance indicators, or monetary incentives, a lack of perceived appreciation was noted. This was especially associated with corporatisation and a hierarchy that at times seem to overlook the important motivators that do drive veterinary professionals. The AVA’s wellness report similarly identified: “Vets do not do their job for money.” (13). Veterinary professionals face unique ethical dilemmas which combine to create an emotionally charged work environment. Participants disclosed feelings that arise when they cannot proceed with life-saving treatment without first having to effectively ask: “how much is your pet’s life worth to you?” (Louise). Veterinarians and vet nurses are often constrained in how they may act when there is a conflict between their obligations and duties to the animal patient and the animal owner. The conflict between the individual’s own moral standards and their professional obligations may create irreconcilable moral and ethical dilemmas (12).

Perspectives about suicide prevention in the veterinary health sector focused on the need for gatekeeper-style suicide prevention training across the profession. Further, participants emphasized the need for increased resources, including the quality, availability of, and access to resources for (peer) help-seekers. They also highlighted the importance of having increased knowledge of referral/help resources to assist them in supporting the safety of their peers. It was further acknowledged that suicide prevention would be enhanced by a focus on addressing and improving the factors that contribute to the high rates of suicide from the outset, including poor workplace conditions, poor work/life balance, low pay, and emotional and moral demands of the work environment.

### 4.4 Implications

Taken together, these valuable findings offer some suggestions for the delivery of ASIST to the veterinary profession. This study has implications for workplace training within the veterinary profession globally. The findings provide a foundation for further exploration and more nuanced attendance to training delivery of the ASIST workshop for this profession, which could enhance employee capability to help their peers in suicidal distress in a profession already known to be at risk of suicide.

Several recommendations have come out of this exploratory study. Firstly, related to training delivery, it is suggested that preparatory elements that enhance participants’ readiness to engage in potentially confronting workshop content, especially in regard to self-disclosures and participation in practice sessions, be considered. This may for example, take the form of pre-reading or pre-workshop activities that refer to the importance of self-reflection and awareness of ones own experiences of suicidality (lived experience of suicide, ideation, behavior, etc.), and appropriateness and safety of language and also, support for comfort around such discussions. Equally, consideration should be given to the potential for veterinarians’ hesitation about taking on the helper role, despite their motivation to help their peers. In our study, this hesitation related to lack of knowledge and confidence in potential help services/resources that might be available for referring their peers to. Such feelings and experiences should be validated by the workshop facilitators who could potentially provide first-hand localized knowledge of available resources within the community of concern. This may be of specific relevance given the lack of suicide prevention/mental health knowledge of most veterinarians who do not traditionally have professional qualifications in suicide prevention or mental health. It follows that they may therefore have limited experience of related resources and referral options. In our study, participants felt that the ASIST model provided great ‘initial knowledge’ but that they wanted to know more about ‘what comes next’. Therefore, providing knowledge about warm referrals/handover process using local resources and processes may be of benefit. Next steps for research should be to design more rigorous evaluation of ASIST for veterinary professionals, as well as to explore workplace supports, culture, and appreciation of factors likely to contribute to experiences of distress within the veterinary profession.

## 5 Limitations

The qualitative approach and the small number of participants in the current study enabled the researchers to collect rich and detailed data. However, as with all qualitative research, the results cannot necessarily be generalized to the whole population, and each individual’s experience will be unique to their own life experiences and view of the world and their role within their profession/workplace. Nevertheless, the data collected provided valuable insights into the experiences of those who participated. Finally, a post-workshop interviewing timeframe of 2 weeks was set by the researchers, however, some interviews were conducted up to 4 weeks post workshop to accommodate individual schedules. Therefore, the potential for delayed or inaccurate recall is a possibility.

## 6 Conclusion

The current study could be interpreted as an exploratory pilot study which investigated veterinarians’ perceptions of ASIST training for their profession.

Participants in this study unanimously agreed that the number of suicides within their profession is unacceptable and that suicide prevention responses on multiple levels are warranted for their profession. After experiencing the ASIST workshop, participants perceived that ASIST was valuable and useful, and expressed a desire for ASIST training to be delivered across the entire veterinary profession in Australia. Training was perceived to have been enhanced by the profession-specific nature of its delivery (including having a veterinarian facilitator). Specifically, a remarkable and impactful aspect of the training was the shared experience of peers and colleagues.

Findings supported future delivery of the ASIST workshop to the veterinary profession, with suggestions made for improvements as well as nuances for targeting profession-specific needs within the training. However, participants also identified potential barriers including cost and time out of the workplace for participation.

Finally, our study highlighted important directions for suicide prevention within the veterinary profession in Australia including ongoing focus and workplace actions required to improve mental health in the workplaces of veterinarians. Further, ASIST was a well-received and useful resource to support this goal, especially if funding became available for its delivery in work time.

## Data Availability

The datasets presented in this article are not readily available because only group data will be available—apart from specific quotes selected from the larger group data for the publication. Requests to access the datasets should be directed to judith.smith2@alumni.griffithuni.edu.au.
